# Cardiovascular disease in India: Lessons learnt & challenges ahead

**Published:** 2010-11

**Authors:** Dorairaj Prabhakaran, Salim Yusuf

**Affiliations:** Center of Excellence in CArdio-metabolic Risk Reduction in South Asia (CoE-CARRS) Public Health Foundation of India (PHFI) & Center for Chronic Disease Control (CCDC) C-1/52, 2^nd^ Floor Safdarjung Development Area New Delhi 110 016, India. dprabhakaran@ccdcindia.org; * Population Health Research Institute McMaster University Hamilton Health Sciences Hamilton, Ontario, Canada. yusufs@mcmaster.ca

Lifestyles of populations across the world have changed dramatically in the 20^th^ century. These changes (collectively termed as epidemiological transition) have been brought about by a number of developments in science and technology that now affect every facet of human existence. Most human societies have moved from agrarian diets and active lives to fast foods and sedentary habits. Combined with increasing tobacco use, these changes have fuelled the epidemic of obesity, diabetes, hypertension, dyslipidaemia and cardiovascular diseases (CVD).

In developed nations the rise in the burden of CVD occurred over several decades due to a long period of epidemiological transition. In India, perhaps because of the rapid pace of economic development, epidemiological changes have spanned a much shorter time. As a consequence, cardiovascular disease (CVD) has emerged as the leading cause of death all over India, with coronary heart disease (CHD) affecting Indians at least 5-6 years earlier than their western counterparts^1^,^2^. Current estimates from disparate cross-sectional studies indicate the prevalence of CHD to be between 7-13 per cent in urban and 2-7 per cent in rural India^3^. The spiralling rates of modifiable risk factors for CHD across the spectrum of rural to urban segments of our population have been demonstrated by several studies across India^3^,^4^. In addition, migration and urbanization have resulted in an increase in the prevalence of risk factors such as diabetes, overweight^5^.

The economic impact of these transformations was estimated at 9 billion dollars in national income from premature deaths due to heart disease, stroke and diabetes in 2005 alone, with the projected estimates of 237 billion dollars by 2015. The out-of-pocket health expenses incurred by households increased from 31.6 per cent in 1995 to 47.3 per cent in 2004^6^. Modelling studies have estimated that if non-communicable diseases (NCDs) were completely eliminated, the estimated GDP in a year would have been 4-10 per cent higher^7^.

The determinants and the control strategies of the CHD epidemic are multi-factorial, complex and interrelated (Fig.). We now know that the surge in CHD and their risk factors in varied settings are fuelled by modifiable risk factors which can be reduced by simple strategies. Most of these preventive strategies are in the domain of policy, health system intervention, health promotion and simple quality improvement programmes aimed at improving secondary prevention.

**Fig:**
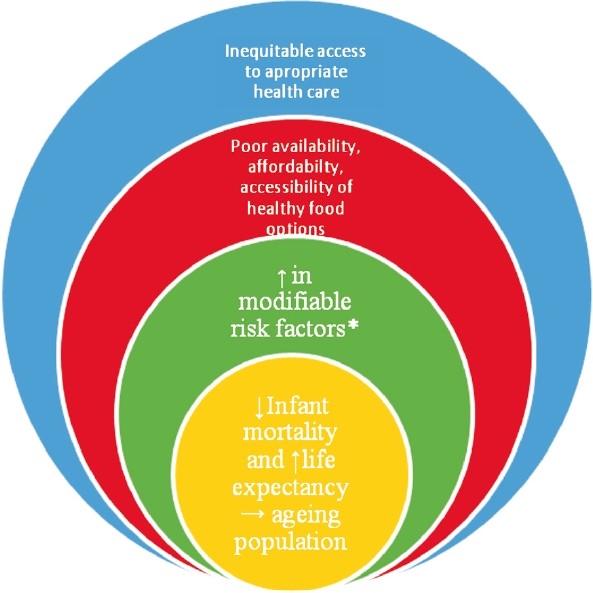
The determinants and the control strategies of the coronary heart disease epidemic. *Established modifiable risk factors include smoking, alcohol, physical inactivity, low consumption of fruits and vegetables, overweight and obesity, increased blood pressure, impaired fasting glucose and dyslipidaemia.

While the need for research directed towards implementing simple but effective CVD prevention strategies and health system strengthening to combat CVD is glaringly obvious, there is very little actual research output in these areas from India^8^,^9^. Key research areas to combat CVD should include: (*i*) cost-effective, innovative ways of reducing CVD risk through health policy and health system interventions; (*ii*) methods for ensuring integration of CVD care within health systems; (*iii*) health system financing strategies for individuals with CVD; (*iv*) best methods of applying existing knowledge for development, implementation and evaluation of CVD prevention programmes; (*v*) mechanistic research to identify the reasons for the younger age of onset of CVD and diabetes and their occurrence at a lower threshold of risk factors; and (*vi*) methods to implement health promotion measures to the population at large along with formulation and implementation of ‘HEART-friendly’ policy measures. The above described measures require multidisciplinary, multi-sectoral and multi-level co-ordination and action. Thus translational research, (both T1: from the laboratory to studies in humans and T2: from clinical research to clinical practice and beyond) is needed to develop multi-pronged approaches that address the patient, provider, healthcare systems, public health, and public policy for the prevention and control of CHD in India.

## What does this issue contribute?

This special section is an attempt to highlight current research trends in the field of CHD and to identify gaps in knowledge. The contributors to this issue are key researchers in India with years of experience in CVD prevention and clinical care. They bring their personal insights, deliberate on the current scenario and identify future areas of research in CVD. The topics cover important areas that include epidemiology, genetics and clinical management with a special focus on the Indian context. These are specially written to provide important information for researchers and clinicians. We hope these articles will help stimulate future researchers and assist in planning their studies. We are extremely grateful to the authors who have taken time from their busy schedules and contributed to this issue.
